# Fiber Optical Hydrogen Sensor Based on WO_3_-Pd_2_Pt-Pt Nanocomposite Films

**DOI:** 10.3390/nano11010128

**Published:** 2021-01-08

**Authors:** Jixiang Dai, Yi Li, Hongbo Ruan, Zhuang Ye, Nianyao Chai, Xuewen Wang, Shuchang Qiu, Wei Bai, Minghong Yang

**Affiliations:** 1 National Engineering Laboratory for Fiber Optic Sensing Technology, Wuhan University of Technology, Wuhan 430070, China; djx409081947@163.com (J.D.); 1904719@student.uwtsd.ac.uk (Y.L.); kingrhb@163.com (H.R.); zhuangye1107@163.com (Z.Y.); scqiu@whut.edu.cn (S.Q.); baiwei@hbtcm.edu.cn (W.B.); 2 Foshan Xianhu Laboratory of the Advanced Energy Science and Technology, Guangdong Laboratory, Foshan 528216, China; Nianyaochai@whut.edu.cn; 3 State Key Laboratory of Advanced Technology for Materials Synthesis and Processing, Wuhan University of Technology, Wuhan 430070, China

**Keywords:** WO_3_-Pd_2_Pt-Pt nanocomposite films, gasochromic properties, InGaAs photoelectric detectors

## Abstract

In this paper, WO_3_-Pd_2_Pt-Pt nanocomposite films were deposited on a single mode fiber as the hydrogen sensing material, which changes its reflectivity under different hydrogen concentration. The reflectivity variation was probed and converted to an electric signal by a pair of balanced InGaAs photoelectric detectors. In addition, the performance of the WO_3_-Pd_2_Pt-Pt composite film was investigated under different optical powers, and the irrigating power was optimized at 5 mW. With the irrigation of this optical power, the hydrogen sensitive film exhibits quick response toward 100 ppm hydrogen in air atmosphere at a room temperature of 25 °C. The experimental results demonstrate a high resolution at 5 parts per million (ppm) within a wide range from 100 to 5000 ppm in air. This simple and compact sensing system can detect hydrogen concentrations far below the explosion limit and provide early alert for hydrogen leakage, showing great potential in hydrogen-related applications.

## 1. Introduction

Hydrogen is known as the next generation of clean energy [1,2], with the potential to replace fossil fuels. However, hydrogen is very dangerous due to its highly flammable characteristics and the smallest molecular size. Moreover, its explosion limit covers a wide range, of 4–75% (volume percent, in air). Hydrogen leakage can lead to enormous casualties and damage to related facilities [3]. Therefore, it is very important to detect hydrogen concentration in real-time by employing a reliable and intrinsically safe hydrogen sensor. Commercial electrochemical hydrogen sensors can be explosive due to potential electric sparks [4,5]. Optical fiber hydrogen sensors utilize an optical fiber carrying weak optical signal, which can avoid applying electrical signals in a flammable and explosive atmosphere. Another advantage of optical fiber hydrogen sensors is that they are less sensitive to electromagnetic noise than electrochemical hydrogen sensors [6]. Therefore, optical fiber hydrogen sensors have drawn great research interest due to their excellent characteristics [7,8], such as their safety, small size and immunity to electromagnetic interference.

Several kinds of optical fiber hydrogen sensors, such as a micro-mirror sensor [9,10], surface plasmon resonance (SPR) sensor [11,12], evanescent wave sensor [13,14] and fiber Bragg grating sensor [15,16,17,18] have been proposed and investigated in recent years. Among these sensors, the micro-mirror hydrogen sensor has a simple structure, tiny size and low cost, making it a good candidate for monitoring hydrogen leakage in air. However, the performance of the micro-mirror hydrogen sensor is easily interfered with by the intensity fluctuation of the optical sensing system, which reduces its sensing accuracy. To overcome this drawback, a compact fiber optical hydrogen sensing system is proposed in this paper. Two InGaAs photoelectric detectors were utilized to reduce the fluctuation effect from the light source. The first InGaAs photoelectric detector (PD1) is used to monitor the partial optical power of the light source (I_1_), while the other (PD2) is employed to measure the optical power reflected by sensing probe (I_2_). By using I_2_/I_1_ as the sensing signal, the fluctuation of the sensing system can be greatly compensated. In addition, WO_3_-Pd_2_Pt-Pt nanocomposite films were deposited on a fiber tip to improve the responsibility of the sensing probe. The WO_3_ thin film was used as the basal layer, as it can display excellent hydrogen-induced discoloration ability when Pd [10] or Pt [19,20] is used as a catalyst. Pd has a good selectivity toward hydrogen [15,16], and it is utilized as the main composition of the catalyst layer. However, hydrogen sensors based on pure Pd films easily suffer from the Pd film’s fatal fracture, caused by its α-β phase transition [16]. By alloying Pd with other metals, such as Au [21,22] and Pt [23], the structural stability of hydrogen sensitive films can be significantly improved. The Pt thin film was selected as the protective layer, which is mainly due to its excellent antioxidant capacity [24]. The proposed system effectively improved the performance of fiber optic hydrogen sensing, which shows its great potential in many related fields.

## 2. Materials and Methods

First, the polymer coating of the single mode fiber (9/125 μm, YOFC Inc., Wuhan, China) was removed by a wire stripper. Subsequently, the bare fiber was cut by a fiber cleaver to form a flat section for depositing the sensitive layer. In the following process, a 160 nm thick WO_3_ film was deposited on the tip of a single mode fiber by using a thermal evaporation system (Rankuum Machinery LTD, Chengdu, China). During the evaporation process, oxygen with a flowing velocity of 200 sccm was supplied as process gas to avoid the loss of oxygen atoms in the deposited coating. After this process, the sample was placed in the chamber of a BESTECH sputtering system for the sputtering catalyst layer. Under 0.5 Pa sputtering pressure of Ar, the 40 nm Pd_2_Pt and 5 nm Pt thin films were sputtered on the surface of the WO_3_ film as the catalyst layer. During the depositing process, the thickness of the hydrogen sensitive film was monitored by the quartz crystal method, and the corresponding deposition rates for WO_3_, Pd and Pt were 0.03, 0.10 and 0.05 nm/s, respectively. Meanwhile, coatings on several Si and SiO_2_ substrates were also prepared in the same run for further characterization.

**Figure 1:**
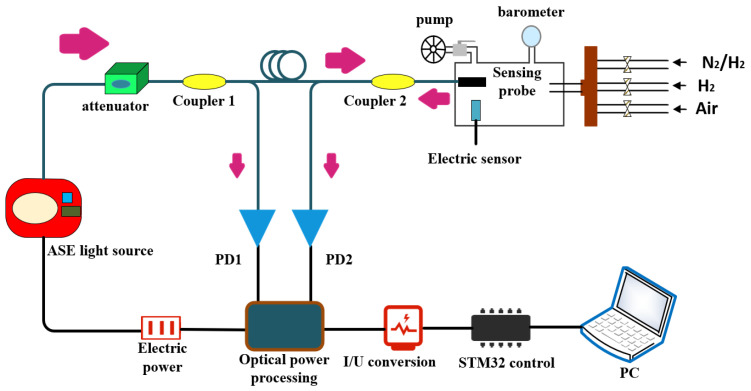
Schematic of the optical fiber hydrogen sensing system.

As it is shown in Figure 1, an optical attenuator is used to connect the optical power of a 13 dBm amplified spontaneous emission (ASE, 1525~1565 nm) light and an optical coupler (20:80). The lower fraction (about 20%) power of the light source is detected by PD1, and the residual power of light source is guided to the sensing probe by a 3 dB (50:50) optical coupler. Electric signals of PD1 and PD2 are used to evaluate the optical power change of the ASE light source and the optical power reflected by the sensing probe, respectively. The hydrogen sensing performance test was carried out at room temperature of 25 °C using air as carrier gas. 1% H_2_ mixed gas (H_2_/N_2_ = 1:99, volume ratio) and 99.99% H_2_ were used as H_2_ supplying gas. 21% O_2_ mixed gas (O_2_/N_2_ = 21:79, volume ratio) was used as dry air for hydrogen sensing. Three mass flow controllers (CS200A, 0~30 sccm, 0~100 sccm, 0~1000 sccm, Beijing Sevenstar, Inc., Beijing, China) were used to control the flowing rate of three gases, respectively. In this work, hydrogen concentrations ranged from 100 to 300 ppm, provided by mixing 1% H_2_ and 21% O_2_ mixed gas with a total flowing rate of 1000 sccm, and hydrogen above 300 ppm was provided by mixing 99.99% H_2_ and 21% O_2_ mixed gas with the same flowing rate. During the hydrogen testing process, the collecting data were recorded by a computer for further analysis.

## 3. Discussion

**Figure 2:**
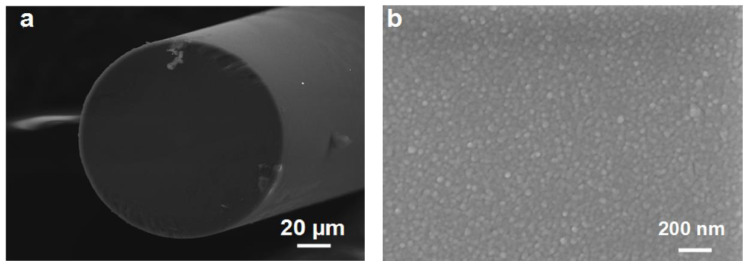

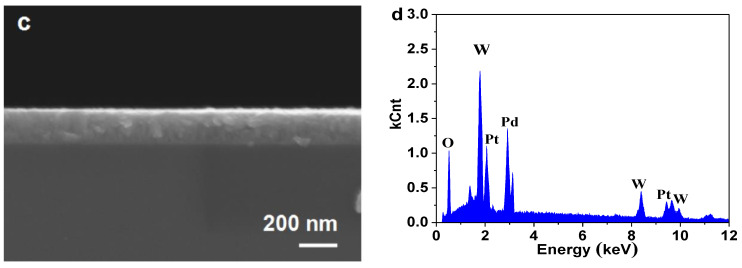
(**a**) SEM image of fiber tip; (**b**) SEM of surface of WO_3_-Pd_2_Pt-Pt composite film; (**c**) SEM of cross section of Si substrate deposited with WO3-Pd2Pt-Pt composite film (**d**) EDAX pattern of WO_3_-Pd_2_Pt-Pt composite film.

The morphology of the hydrogen sensitive film (after hydrogen exposure) was characterized using a field emission scanning electron microscope (FE-SEM ULTRA PLUS-43-13, Zeiss, Germany). As is shown in Figure 2a,b, the surface of the prepared film looks dense and homogeneous after hydrogen exposure. There is no obvious micro-cracking on the surface, demonstrating its good mechanical properties. A cross-section of the Si wafer substrate is displayed in Figure 2c. The total thickness of the WO_3_-Pd_2_Pt-Pt composite film is about 200 nm, which is consistent with the setting value. The WO_3_ thin film has a good adhesion to the optical fiber [10,18], making it an ideal basal layer. The Pd-Pt composite film has a better stability and catalytic ability than pure Pd [25]. Therefore, the hydrogen sensitive film has a good stability during hydrogen exposure. The elemental analysis was carried out by energy dispersive X-ray analysis (EDAX) using an X-ray detector attached to the FE-SEM instrument. As displayed in Figure 2d, the molar ratio of W, Pd and Pt is about 9:6:4, which is approximately consistent with the actual W:Pd:Pt of 160 nm WO_3_, 40 nm Pd_2_Pt composite film and 5 nm pure Pt film.

**Figure 3:**
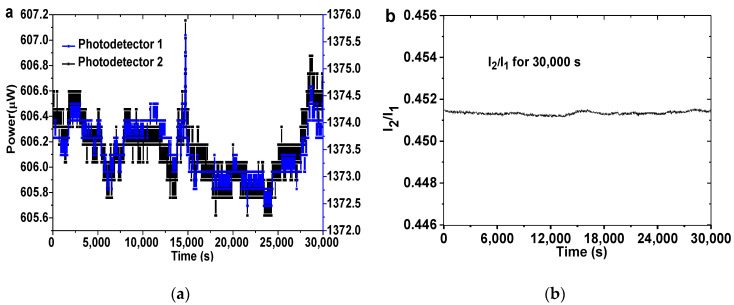
(**a**) Reference intensity (I_1_) and sensing intensity (I_2_); (**b**) the balanced signal I_2_/I_1_; collecting duration for 30,000 s at a room temperature of 25 °C.

Figure 3a gives the change of reference intensity (I_1_) and sensing intensity (I_2_) for 30,000 s at a room temperature of 25 °C, and the fluctuation of I_1_ and I_2_ is about 0.3% of their initial value. The variation tendency of the reference intensity is nearly the same as that of the sensing intensity, which could be attributed to the optical intensity fluctuation caused by the light source. However, the fluctuation of the intensity ratio (I_2_/I_1_) is less than 0.0003 (in Figure 3b), which is about 0.07% of I_1_/I_2_. Therefore, the signal noise ratio of the hydrogen sensing system can be remarkably improved by using I_2_/I_1_ as measurement parameters.

The sensing principle of this sensor is based on the hydrogen-induced gas-chromic effect of the WO_3_-Pd_2_Pt-Pt composite film. Although PdH_x_ will be produced during hydrogen exposure, the reflectance change of the sensing probe caused by WO_3−x_.xH_2_O [19,25] is much greater than that of PdH_x_ [9]. Therefore, the main sensing mechanism between the nanocomposite film and H_2_ can be expressed in the following two reaction equations. The reflectance change of the nanofilm can be attributed to absorptions of photons involving the defect band [26], which is caused by the coexistence of the water molecules and oxygen vacancies in its band gap during the hydrogen response.

(1) $${\mathrm{WO}}_{3}+{\mathrm{xH}}_{2}\overset{\mathrm{Catalyst}}{\to }{\mathrm{WO}}_{3-x}{.\mathrm{xH}}_{2}O$$

(2) $${\mathrm{WO}}_{3}{}_{-x}{.\mathrm{xH}}_{2}O+\frac{x}{2}O_{2}\overset{\mathrm{Catalyst}}{\to }{\mathrm{WO}}_{3}+{\mathrm{xH}}_{2}O$$

**Figure 4:**
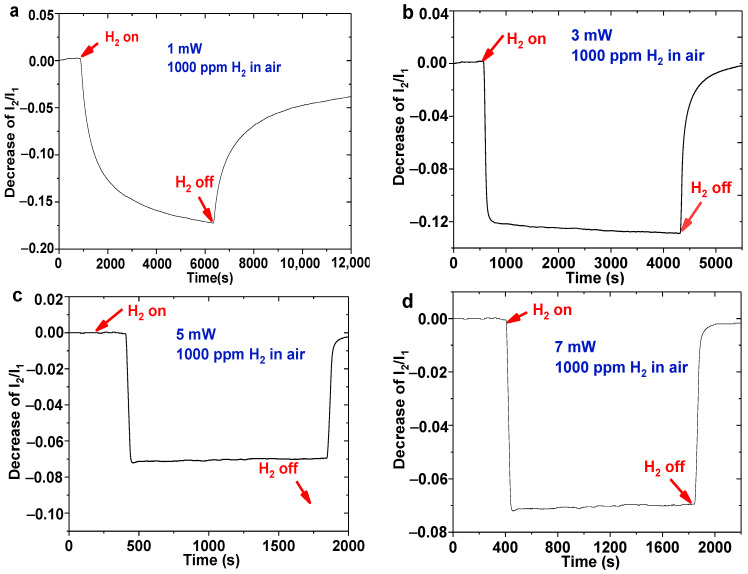
Hydrogen response of sensing probe irrigated by different optical powers: (**a**) 1 mW; (**b**) 3 mW; (**c**) 5 mW; (**d**) 7 mW.

To investigate the sensing performance of the WO_3_-Pd_2_-Pt composite film, an optical attenuator was used to adjust the optical power reaching to the fiber tip. Figure 4a gives the response of the sensing probe during 1000 ppm hydrogen exposure when irrigated at 1 mW. It can be seen that I_2_/I_1_ decreases faster at the beginning, then gradually slows down, and does not reach an equilibrium even after 5000 s. Moreover, the bleaching process is also much slower when the sensing probe is exposed to air under this irrigating power. When the power increases to 3 mW, 5 mW and 7 mW, the I_2_/I_1_ decreases sharply and almost reaches an equilibrium in the first ten seconds of the hydrogen response.

In addition, the hydrogen sensor displays a quicker response rate and lower sensitivity with an increasing irrigating power. The reason for this phenomenon may be that the hydrogen reaction of this sensitive film is an exothermic reaction [17,20]. Therefore, it is easier to reach a reaction equilibrium with the heating of the light source. It can also be observed that the sensing probe shows a better stability and responsibility with the irrigating power of 5 mW. To ensure this hydrogen sensor with higher sensitivity and short response time, 5 mW is a relatively ideal irrigating power. Further hydrogen measurements of the sensing probe will be carried out at this condition.

As for hydrogen sensors based on metallic or metal-oxide films, the stability of these sensors is closely related to the responsibility of sensitive materials. As reported, FBG coated with a 350 nm Pd film without laser heating has no response to 10% H_2_ at an operating temperature of −50 °C [27]. Hydrogen cannot penetrate into sensitive film at a low operating temperature, resulting in the poor responsibility and stability of the sensor. A hydrogen sensor based on WO_3_ can display lower sensitivity at much higher working temperatures because the response between sensitive material and hydrogen is an exothermic reaction [17]. Moreover, the hydrogen sensitive film may react with other reducing gas at a higher working temperature [28], leading to the poor selectivity of the sensor. Therefore, a feasible method to improve the stability of sensor is to keep the film working at the proper temperature [29,30], which can ensure the responsibility and anti-interference ability of the sensitive film.

**Figure 5:**
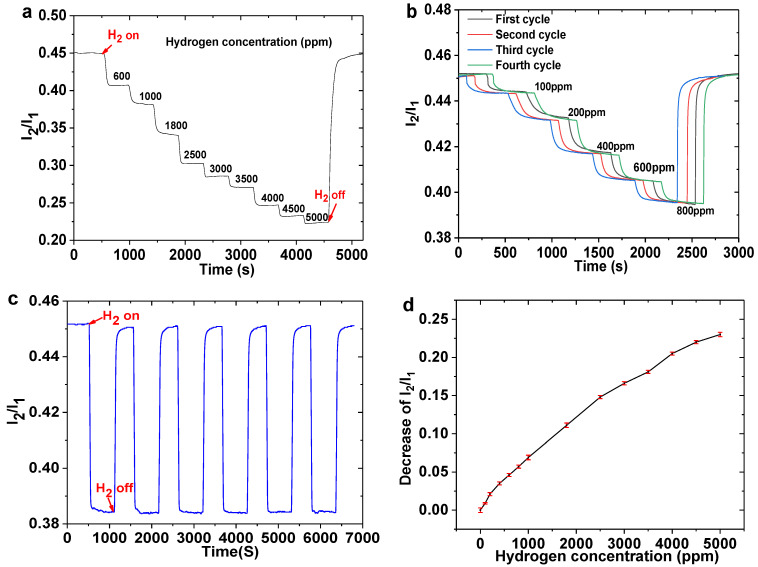
(**a**) Hydrogen response of sensing probe with continuous increase of hydrogen concentrations; (**b**) Four cycles of hydrogen sensing probe under different hydrogen concentrations; (**c**) six cycles of 1000 ppm hydrogen exposure; (**d**) Decrease of I_2_/I_1_ under different hydrogen concentrations.

Figure 5a illustrates the hydrogen response of the sensing probe under the continuous increase of hydrogen concentrations. When the hydrogen concentrations are 600, 1000, 1800, 2500, 3000, 3500, 4000, 4500 and 5000 ppm, the decreases of I_2_/I_1_ are 0.0463, 0.0694, 0.1117, 0.1482, 0.1668, 0.1812, 0.2054, 0.2207 and 0.2316, respectively. As shown in Figure 5b, the hydrogen sensor shows good repeatability during the four cycles of 100, 200, 400, 600 and 800 ppm hydrogen exposure, and the corresponding decreases of I_1_/I_2_ are about 0.0096, 0.0215, 0.0357, 0.0462 and 0.0573 respectively. Since the fluctuation of I_2_/I_1_ in several seconds is less than 0.0002, the hydrogen resolution of this sensing probe can reach 5 ppm within the range of 100 to 5000 ppm.

The response time is calculated from the hydrogen flowing into the gas chamber to I_2_/I_1_ reaching 90% of the total increase, while the recovery time starts as the sensing probe is exposed to the air and ends when I_2_/I_1_ achieves 90% of decrease. The proposed sensor responds immediately when hydrogen is introduced into the gas chamber. Generally, this sensor displays a quicker response rate toward higher concentrations of hydrogen. The response time of this sensor is about 20 s when hydrogen concentration exceeds 1000 ppm. When hydrogen concentration decreased to 100 ppm, the response time of this sensor nearly doubled. The recovery time of this sensor is also tens of seconds. This is mainly due to the insufficient diffusion power of low-concentration hydrogen at room temperature, and it takes longer to reach the reaction equilibrium.

As displayed in Figure 5c, the hydrogen sensor shows a good reproducibility during six cycles of 1000 ppm hydrogen exposure, indicating the good stability of this sensing system. Figure 5d shows the decrease of I_2_/I_1_ under different hydrogen concentrations. The hydrogen sensor shows a non-linear hydrogen response, which is similar to that of the reported work [21,22]. The error bars of different hydrogen concentrations illustrate the fluctuations of I_2_/I_1_ during the hydrogen testing process. The hydrogen sensor shows better sensitivity toward lower hydrogen concentrations. The reason for this phenomenon is the excellent gaschromic properties of the WO_3_-Pd_2_Pt-Pt composite film, the optimization of the irrigating power and the signal processing method. The response and recovery times of this sensor are comparable to those of our previous work [31,32], and comparisons with other reported works [33,34,35,36,37,38] are also presented in Table 1. Although the response rate of this sensor is not outstanding, it can give us a clue on how to prepare an optical fiber hydrogen sensor with a high sensitivity and low cost. Further improvements can be achieved by optimizing the composition and working temperature of sensitive materials.

## 4. Conclusions

A simple and compact optical fiber sensing system, which is based on a WO_3_-Pd_2_Pt-Pt composite film and two InGaAs photoelectric detectors, is proposed and has been experimentally investigated in this paper. Under an optimized irrigating power of 5 mW, the resolution of the proposed hydrogen sensor can reach 5 ppm when the hydrogen concentration ranges from 100 to 5000 ppm. Theoretically, the detection limit of this hydrogen sensor can be as low as 10 ppm at room temperature. Moreover, this hydrogen sensing system shows good repeatability during the hydrogen exposure process. The proposed hydrogen sensing system is very promising for hydrogen leakage warning in air.

## Figures and Tables

**Table 1:** Comparison of several types of optical hydrogen sensor.

Publication Year, Reference	Configuration of Sensing Head, Testing Environment	Concentration Range, Sensitivity or Wavelength Shift, Response Time, Operating Temperature	Cost of Sensing System
2008, [17]	FBG + LPFG, Pt-loaded WO_3_ coating, air	0.6%–4%, 1.2–8 nm, 4 s, 25 °C (FBG + 15 dB LPFG)	High
2015, [33]	15 nm Pd + 3.3 μm MFBG, N_2_	−1.08 nm wavelength shift 5%, 60 s, room temperature	Moderate
2018, [34]	Pt-loaded WO_3_/SiO_2_ coating + PDMS double C cavity, air	0–1%, about 12.5 ppm; 23 s, room temperature	High
2019, [35]	Nanofiber enhanced stimulated Raman spectroscopy, N_2_	0–4%, several ppm; less than 10 s, room temperature	High
2019, [36]	Pd film deposited on PDMS substrate, N_2_	0–4%, about 55% reflectance decrease (4%H_2_/N_2_); more than 20 s, room temperature	High
2020, [37]	Pt loaded WO_3_/SiO_2_ coating+polymer planar Bragg grating	0–0.2%, 5 ppm; tens of seconds, room temperature	High
2020, [38]	Ge/P doped fiber, crude oil	2.24 psi, 1 mol m^−3^, about ten days, 300 °C	Moderate
This work	WO_3_-Pd_2_Pt-Pt film deposited on single mode fiber, air	0.01%–0.5%, 5 ppm; 20 s (above 0.1%H_2_), room temperature	Low
